# Dissecting Sex‐Specific Pathology in K18‐hACE2 Transgenic Mice Infected With Different SARS‐CoV‐2 Variants

**DOI:** 10.1002/jmv.70506

**Published:** 2025-07-21

**Authors:** Elysia A. Masters, Weichun Tang, Insung Kang, Martina Kosikova, Jennifer H. Hanks, Lana Elkins, Hyung‐Joon Kwon, Uriel Ortega‐Rodriguez, Binsheng Gong, Kelly E. Mercer, Hang Xie

**Affiliations:** ^1^ Division of Systems Biology, National Center for Toxicological Research United States Food and Drug Administration Jefferson Arkansas USA; ^2^ Division of Viral Products, Office of Vaccines Research and Review, Center for Biologics Evaluation and Research United States Food and Drug Administration Silver Spring Maryland USA; ^3^ Toxicologic Pathology Associates Jefferson Arkansas USA; ^4^ Division of Neurotoxicology, National Center for Toxicological Research United States Food and Drug Administration Jefferson Arkansas USA; ^5^ Division of Bioinformatics and Biostatistics, National Center for Toxicological Research United States Food and Drug Administration Jefferson Arkansas USA

**Keywords:** COVID‐19, K18‐hACE2 transgenic mouse, pathology, SARS‐CoV‐2, sex differences, spatial transcriptomics

## Abstract

Sex‐biased differences in COVID‐19 outcomes in relation to individual SARS‐CoV‐2 variants are not well understood. In this study, lungs and nasal cavities of age‐matched female and male K18‐hACE2 transgenic mice were collected for dissecting sex‐specific differences in pathology after infection of SARS‐CoV‐2 614 G, Delta, or Omicron variant. Overall, Delta infection induced the most severe inflammation and pathology in nasal cavity and lung followed by the 614 G, then Omicron variant. Sex differences in host responses to SARS‐CoV‐2 infection were variant‐specific. Delta‐infected males showed increased pulmonary infiltration of CD163+ “M2” macrophages, Ly6G+ neutrophils, and NKR‐P1C + NK cells during early onset of infection, and elevated lung inflammatory cytokines such as IL‐10, IL‐6, and IP‐10 than Delta‐infected females. Conversely, females had increased lung CD4 + T cell recruitment after Omicron infection and significantly elevated lung MCP‐1 secretion after Delta infection than males. Lung spatial transcriptomics data revealed that Delta‐infected females had enriched gene pathways related to humoral immune response and interferon signaling, while males had enriched pathways associated with extracellular matrix production, chemokine signaling, and cell chemotaxis. Taken together, this study highlights the complex infection dynamics with respect to individual SARS‐CoV‐2 variants and underscores the importance of sex as a confounding factor for COVID‐19 pathology.

## 1 Introduction

Since the outbreak of coronavirus disease 2019 (COVID‐19) pandemic, severe acute respiratory syndrome coronavirus‐2 (SARS‐CoV‐2) has caused over 776 million human infections and 7.1 million deaths worldwide, of which 103 million cases and 1.2 million deaths were reported in the U.S. alone as of November 1, 2024 (COVID‐19 cases | WHO COVID‐19 dashboard). As our understanding of SARS‐CoV‐2 pathogenesis evolves, significant disparities in disease severity and outcomes have been identified, which are associated with demographic locations, socioeconomic status, race, ethnicity, age, and/or sex [1, 2, 3, 4, 5, 6, 7, 8, 9]. Sex differences in immune responses to SARS‐CoV‐2 have been found to influence the progression and severity of COVID‐19 [6, 10, 11, 12, 13]. It is reported that male patients often exhibited a heightened innate immune response while female patients usually responded with more robust T cell activation following SARS‐CoV‐2 infection [6, 10, 11, 12, 13]. Correspondingly, men often are reported to experience more severe COVID‐19 outcomes including longer hospitalization stay and higher fatality rate than women, especially during the early pandemic when COVID‐19 vaccines/antivirals were unavailable [8, 14, 15, 16].

As SARS‐CoV‐2 is continuously evolving, mutations have persistently accumulated in the viral spike protein, a surface glycoprotein that not only engages in receptor binding to human angiotensin converting enzyme 2 (hACE2) but also is responsible for viral antigenicity [17, 18, 19, 20, 21]. Many variants with antigenic changes have emerged and have caused different waves of the COVID‐19 pandemic, including 614 G, Alpha, Beta, Gamma, Delta, and Omicron [22]. It remains unclear how these different SARS‐CoV‐2 variants may interact with the host immune system to potentially alter the disease course in males and females. The current study was focused on investigating sex‐specific pathology patterns across various SARS‐CoV‐2 variants in K18‐hACE2 transgenic mice. The K18‐hACE2 transgenic mouse strain is a preclinical animal model originally established for studying SARS‐CoV‐1 pathogenesis [23], but has been instrumental in revealing the complex host immune responses to SARS‐CoV‐2 due to its ability to recapitulate the major COVID‐19 manifestations in humans [24, 25, 26, 27]. In this study, age‐matched male and female K18‐hACE2 transgenic mice were infected with representative SARS‐CoV‐2 variants, namely 614 G, Delta and Omicron from the early, peak, and later waves of the COVID‐19 pandemic. Nasal cavity and lungs were harvested on 3‐ and 5‐day post infection (dpi) for viral load quantification, cytokine profiling, histopathology, and spatial transcriptomics. The findings indicate distinct sex‐biased pathology features with respect to individual SARS‐CoV‐2 variants and underscore the importance of considering sex as a critical variable for studying the pathogenic mechanisms and developing effective countermeasures to treat COVID‐19.

## 2 Methods

### 2.1 Cells and Viruses

Vero E6 stably transfected with human TMPRSS2 (TMPRSS2‐E6) (BPS Bioscience #78081) was maintained in Gibco high‐glucose Dulbecco's modified Eagle's medium (DMEM) supplemented with 10% fetal bovine serum (FBS), 1% penicillin/streptomycin and 10 mM HEPES pH 7.3 plus 3 mg/mL of Puromycin and 1% Na pyruvate at 37°C, 5% CO_2_.

The seeds of SARS‐CoV‐2 clinical isolates 614 G (ATCC# NR‐53516), Omicron BA.1 (ATCC# NR‐56462), and Delta (B.1.617.2) were obtained through BEI Resources or US Centers for Disease Control and Prevention, respectively [25, 26, 27]. All seed viruses were amplified in TMPRSS2‐E6 cells in DMEM supplemented with 3% FBS [25, 27]. Harvested viruses were titrated using an ELISA‐based 50% tissue culture infectious dose (TCID_50_) method [25, 26, 27]. Aliquoted viruses were secured in a −80°C freezer until use. All work with live SARS‐CoV‐2 isolates was conducted in an animal biosafety level (ABSL) 3 laboratory equipped with advanced access control devices and by personnel equipped with powered air‐purifying respirators.

### 2.2 Mouse Infection

B6. Cg‐Tg (K18‐ACE2)2Primn/J (K18‐hACE2) transgenic mice (Jax Stock No. 034860045) were bred at the FDA White Oak vivarium. Age‐matched male and female K18‐hACE2 mice (6–16 weeks old) were ear‐tagged and randomly assigned at 1:1 ratio per experimental group. Under light isoflurane anesthesia, mice were inoculated intranasally with a pre‐determined lethal dose of 614 G (1000 TCID_50_/50 µL/mouse) [25, 26, 27] or Delta (100 TCID_50_/50 µL/mouse) [25, 27, 28], respectively. Omicron BA.1 was inoculated at a high but nonlethal dose of 5000 TCID_50_/50 µL/mouse because of lack of lethality in K18‐hACE2 mice [25]. Infected mice were humanely euthanized on 3 and 5 dpi, respectively according to the time points established previously [21, 26, 27]. Whole lungs were harvested and were immediately fixed in 10% neutral buffered formaldehyde (NBF) for pathology. Nasal cavities were perfused with 10% NBF first and then fixed in 10% NBF for at least 5 days before pathology. Separate sets of whole lungs and nasal cavities were harvested for viral load and cytokine determination. All animal studies were performed according to the protocols approved by FDA White Oak Vivarium IACUC and were repeated at least 2–3 times.

### 2.3 Tissue‐Specific hACE2 Expression, Viral Load and Cytokine Determination

Harvested nasal turbinate and lungs were homogenized and total RNA was extracted for cDNA synthesis [26]. The copies of hACE2 or viral nucleocapsid (N) in homogenized tissues were determined using QuantiNova SYBR Green PCR kit (Qiagen #208052) in combination of the hACE2 specific primer set (Integrated DNA Technologies, Assay ID: Hs. PT.58.27645939) or 2019‐nCoV RUO Kit (Integrated DNA Technologies #10006713). Quantitative amplification was conducted in Stratagene MX3000p qPCR cycler (Agilent) under the following conditions: 95°C for 120 s, 95°C for 5 s and 60°C for 18 s (50 cycles) [26]. Viral loads based on threshold cycle (Ct) values were calculated and interpolated from a standard curve [26]. Separate sets of lung homogenates were tested for pro‐inflammatory cytokines and chemokines using custom MSD V‐Plex kits (Meso Scale Diagnostic #K152A0H‐2) and a MESOQuickPlex SQ 120 imager according to the manufacturer's instructions.

### 2.4 Histopathology

All histology was performed by Toxicologic Pathology Associates (Jefferson, AR). Fixed lungs were routinely processed and embedded in a paraffin‐based infiltrating media (Formula R®). Fixed nasal cavities were first decalcified in 20% EDTA with 5% sucrose and then trimmed into four transverse sections perpendicular to the plane of the hard palate and nasal septum and embedded into a single paraffin block. Embedded blocks were sectioned in approximately 5‐micron thickness and mounted slides were stained with hematoxylin and eosin (H&E) according to standard histology protocols. Digital images were scanned using an Aperio Scanscope System (Leica Biosystems, Wetzlar, Germany). Histopathology lesions were evaluated blindly by a board‐certified veterinary pathologist and graded for severity according to the following scale: 1 (minimal), 2 (mild), 3 (moderate), or 4 (marked). “Histopathology Sum Score” is defined as the sum of all microscopic findings for each animal (summarized in Supporting Information Tables 1 and 2).

### 2.5 Immunohistochemistry (IHC)

After deparaffinization and rehydration, nasal cavity and lung sections were placed on Autostainer 360 (Thermo Scientific) for IHC staining using commercial antibodies against CD68 (cat#ab283654, 1:200), CD8 (cat#ab209775, 1:500), CD4 (cat#ab183685, 1:1000), Ly6g (cat#ab238132, 1:1000), CD163 (cat#ab182422, 1:200) or NKR‐P1C (cat#ab289542, 1:60), or a rabbit polyclonal antibody raised against SARS‐CoV‐2 nucleocapsid (NP) (1:1000) [26]. Tissue sections were further probed by biotinylated goat anti‐rabbit secondary antibody (Jackson ImmunoResearch Laboratories, 1:200) coupled with ExtrAvidin‐Peroxidase (Sigma‐Aldrich,) and color was developed in liquid DAB (Agilent Technologies). Slides were counterstained with hematoxylin before being scanned and analyzed for the presence of NP‐, CD68 + , CD163 + , CD8+ and CD4 + , Lys6g+ and NKR‐P1C+ cells using the whole tissue automatic analysis and the positive pixel algorithm (Aperio Scanscope System). A secondary analysis was conducted for immune cell populations within defined regions of interest (ROI) showing greater than 35% NP‐staining based on the following input parameters for the positive pixel count algorithm: View Width, 1000; View Height, 1000; Overlap Size, 0; Image Zoom, 1; Mark‐up Compression Type, same as processed image; Compression quality, 30; Classifier Neighborhood, 0; Classifier, None; Class List, none; Hue Value, 0.1; Hue Width, 0.5; Color Saturation Threshold, 0.1; Iwp (High), 225; Iwp (Low) = Ip (High); 114, Ip (Low) = Isp (High), 100; Isp (Low), 0; Inp (High), −1. For each marker, total cell counts are expressed as the percentage of the sum of weak, moderate, and strong positive pixel counts divided by total number of pixels for the whole tissue and regional ROI automatic analysis presented.

### 2.6 Spatial Transcriptomics

Representative formalin‐fixed, paraffin‐embedded (FFPE) lungs from Delta‐infected female and male mice on 3 and 5 dpi were selected for spatial transcriptomics. RNA was isolated from 2 × 20 μm curls from each FFPE tissue using RNeasy FFPE Kit (Qiagen) and characterized using the Agilent 2100 Bioanalyzer and High Sensitivity RNA 6000 Pico assay (Agilent). Only samples with RNA quality in a minimum of DV200 of 50 were subjected to spatial transcriptomics. A 6.5 x 6.5 mm region of interest was selected by a pathologist and the tissue block was trimmed with a razor blade for sectioning. A 5 μm section of each lung ROI was placed within the four capture areas of a 10X Genomics Gene Expression slide. Slides were deparaffinized and rehydrated then stained by H&E. Digital images of the tissue capture areas were obtained with an Aperio Scanscope at 200X magnification and exported as. tif files for downstream analysis. Visium libraries were prepared according to the Visium Spatial Gene Expression for FFPE User Guide (10x Genomics) and were sequenced at Baylor Genomics and RNA Profiling Core using NextSeq. 500/NovaSeq. 6000 (Illumina). Resultant libraries were characterized using the Agilent 2100 Bioanalyzer and High Sensitivity DNA assay (Agilent).

Visium libraries (FASTQ files) were demultiplexed and aligned with Space Ranger (v2.1, 10X Genomics) using the mouse reference transcriptome (mm10 2020‐A reference transcriptome, 10X Genomics). Analysis was primarily performed in RStudio (v1.2.1335; R v4.1.1) using the Seurat packages (v4.0.3). Visium datasets were normalized, scaled, and integrated using the SCTransform package and merged into one object. An unsupervised, shared nearest neighbor (SNN) clustering algorithm was used to embed the data as a Uniform Manifold Approximation and Projection (UMAP). Spatial transcriptomics spots represent 55 mm diameter spots for spatially mapped bulk‐sequencing of all cells within a particular spot and spot clusters were defined using resolution = 0.12 expression profiles and spatial location. Conserved genes were identified within each cluster and used in conjunction with the spatial overlay on H&E image to annotate clusters. Differentially expressed genes within a the ImmuneCell cluster were identified for female versus male samples and Y‐linked genes were removed from downstream analysis (Ex: Eif2s3y, Ddx3y and Kdm5d). Enriched pathways for female and male samples were identified using gene ontology, enrichGO package in R.

### 2.7 Statistical Analysis

Data of 2–3 independent experiments were combined and were subjected to two‐way ANOVA or Mann‐Whitney test as specified in individual figure legends (GraphPad Prism v10.2.3). Viral burden data were log transformed before statistical analysis. A *p* value of < 0.05 was considered statistically significant.

## 3 Results

### 3.1 Viral Burden and Histopathology Across SARS‐CoV‐2 Variants

**Figure 1:**
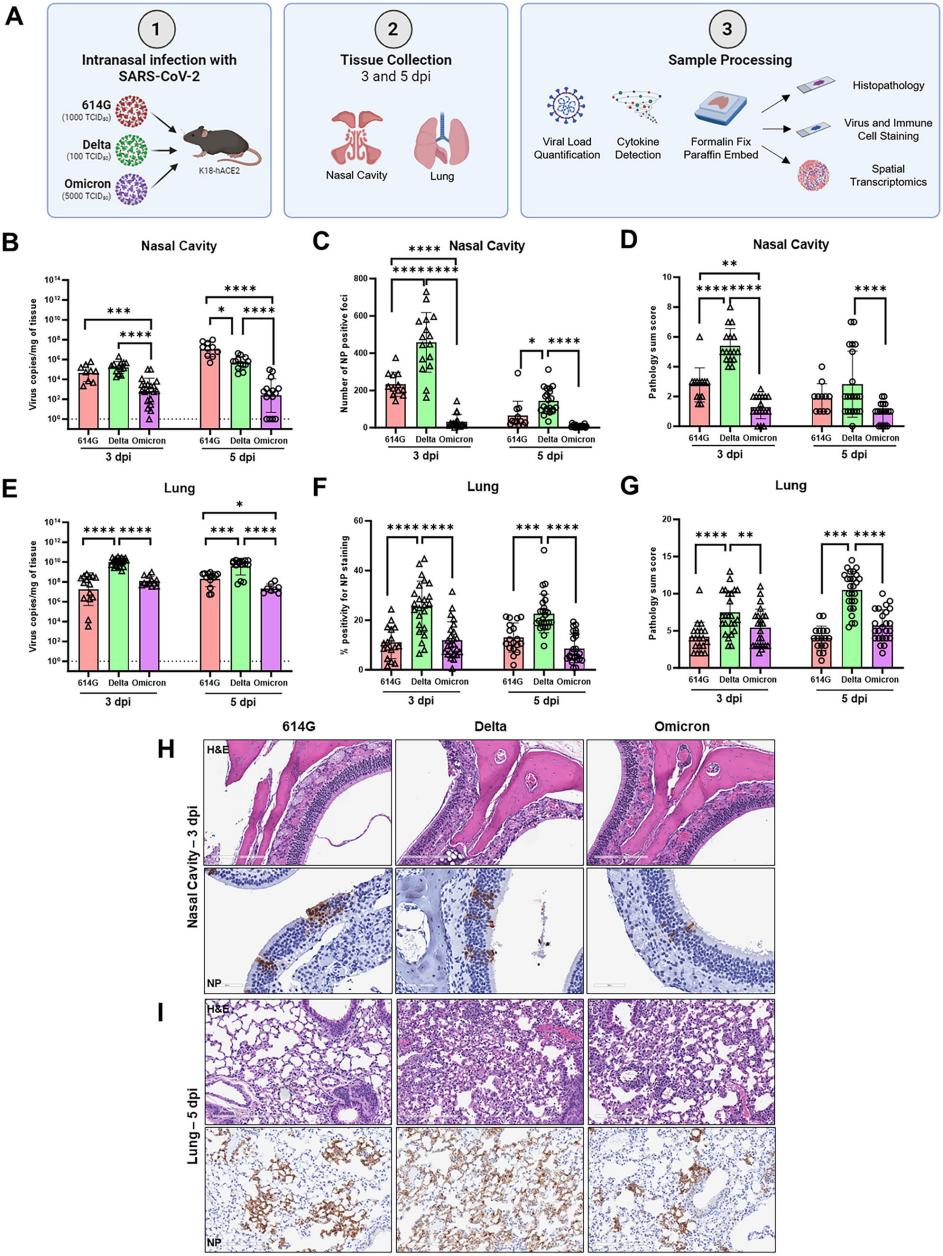
Intranasal infection of K18‐hACE2 mice with SARS‐CoV‐2 variants. (A) Schematic of experimental design outlining infectious doses of 614 G, Delta or Omicron variant, tissue collection timepoints and sample endpoints. Nasal cavity and lungs were harvested on 3‐ and 5‐days post infection (dpi) for virus copies by RT‐qPCR. Tissue sections were also H&E stained for histopathology or IHC stained for viral nucleocapsid (NP). Nasal cavity: (B) virus RNA copies; (C) number of NP positive foci and (D) the histopathology sum scores. Lung: (E) virus RNA copies; (F) percent NP positive staining and (G) the histopathology sum scores. Representative histologic images of infected nasal cavity on 3‐dpi (H) or infected lungs on 5‐dpi (I) are shown (top panels: H&E staining and bottom panels: IHC staining). Results combining 2–3 independent experiments are shown. Individual mouse data are reported with bars/errors indicating either geometric mean ± geometric SD (viral titers) or mean ± SD (pathology scores and NP staining). Dotted line represents RT‐qPCR detection limit. Significance was evaluated by two‐way ANOVA with Sidak's or Tukey's post‐hoc test for multiple comparisons. *p < 0.05, **p < 0.01, ***p < 0.001, ****p < 0.0001.

To assess the impact of SARS‐CoV‐2 infection on nasal cavity and lung, transgenic K18‐hACE2 mice were intranasally infected with 614 G, Delta, or Omicron BA.1 variant. The schematic overview of the experimental design is shown in Figure 1A which outlines infectious doses, tissue collection time points, and sample endpoints. Inoculation dose for each variant was selected based on their ability to induce clinically relevant pathology as determined previously [25, 27]. Despite the inoculation dose for Omicron was 5X of that for 614 G and 50X of that for Delta, both 614G‐ and Delta‐infected mice had significantly higher viral burdens in nasal cavity and lung than Omicron‐infected mice on 3‐ and 5‐dpi (Figure 1B,E). IHC staining confirmed that Delta‐infected nasal cavity and lung sections had the highest levels of viral antigen present followed by 614‐infected tissues than Omicron‐infected tissues on 3‐ and 5‐dpi (Figure 1C,F).

Furthermore, nasal cavity histopathology scores, reflecting the severity of mucosal and respiratory surface degradation were also significantly higher in both 614G‐ and Delta‐infected mice than Omicron‐infected mice on 3‐dpi (Figure 1D). By 5‐dpi, the pathologic severity of infected nasal cavity subsided across all three variants, though Delta‐infected mice maintained higher pathology scores than Omicron‐infected mice (Figure 1D). Histopathological examination of nasal cavity tissues revealed mixed submucosal inflammatory infiltrates, including intranasal exudate and mucosal degeneration in representative H&E images of infected olfactory mucosa on 3‐dpi (Figure 1H). The most pronounced pathological change was observed in Delta‐infected nasal cavity, which the olfactory mucosa in the dorsal meatus was bilaterally denuded, with inflammatory infiltrates and degenerative cellular debris in the nasal lumen (Figure 1H, Top panel middle). A summary of all nasal cavity histopathology findings is included in Supporting Information Table 1.

In lung, Delta‐infected mice had the highest cumulative scores for histopathology on both 3‐ and 5‐dpi (Figure 1G). All infected lung sections, regardless of variant, exhibited different levels of infiltrating mononuclear to mixed inflammatory cells in the alveolar interstitium and alveolar spaces, accompanied by vasculitis/perivasculitis (Figure 1I and Supporting Information Table 2). Notably, bronchiolar tissues were generally spared from inflammatory infiltrates. While a low level of interstitial and alveolar infiltrates was observed across all virus‐infected groups on 3‐dpi, Delta‐infected lungs had the most severe alveolar and interstitial infiltrates and vasculitis/perivasculitis on 5‐dpi (Figure 1I, Top panel middle). These results confirm that Delta is more pathogenic followed by 614 G than Omicron variant.

### 3.2 Sex‐Specific Analysis of Viral Burden and Pathology

**Figure 2:**
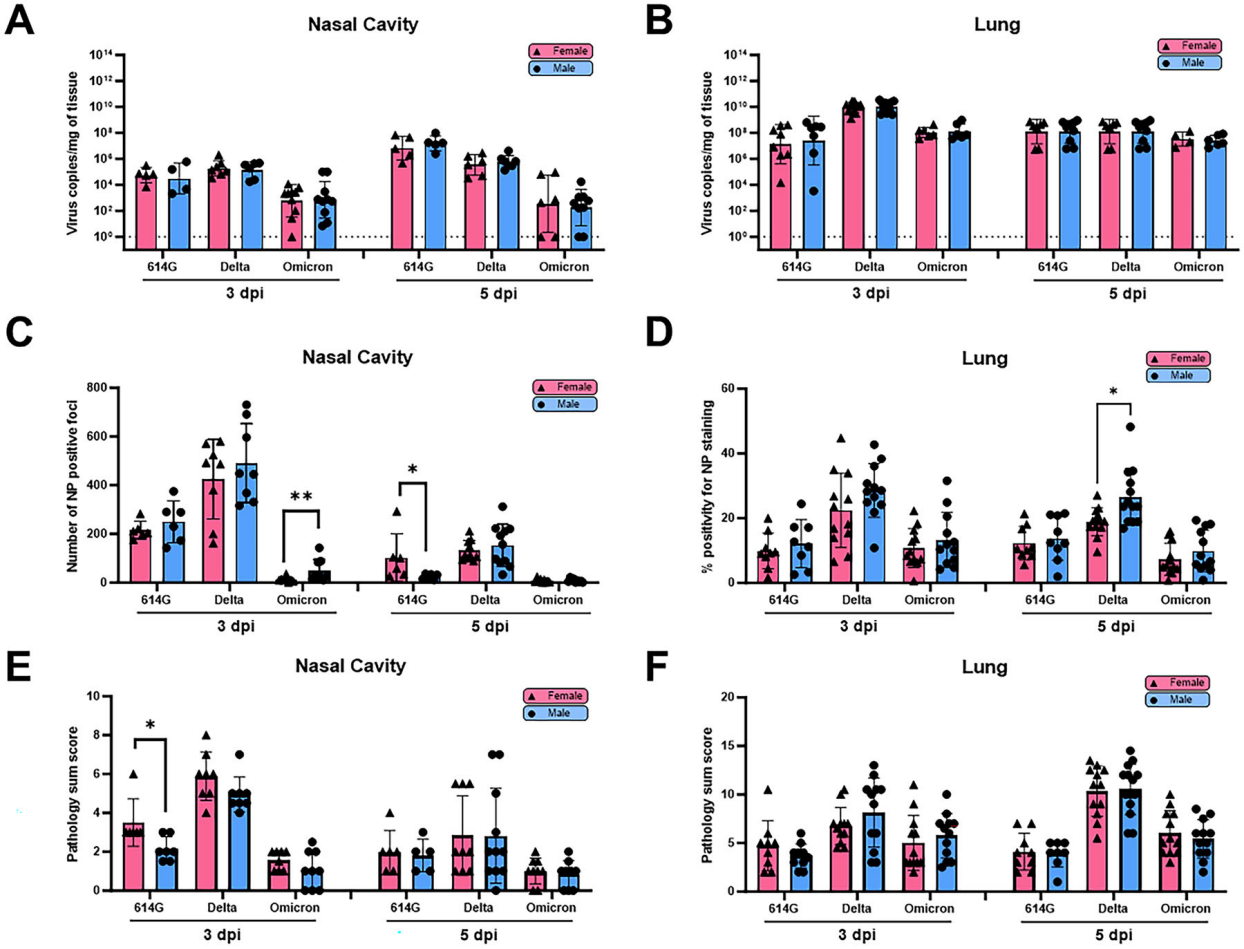
Sex‐specific pathology assessment in infected nasal cavity and lung tissue across SARS‐CoV‐2 variants. Nasal cavity and lungs from age‐matched female and male mice were harvested on 3‐ and 5‐days post infection (dpi) for virus copies by RT‐qPCR. Tissue sections were also H&E stained for histopathology or IHC stained for viral nucleocapsid (NP). Nasal cavity: (A) virus RNA copies; (C) number of NP positive foci and (E) the histopathology sum scores. Lung: (B) virus RNA copies; (D) percent NP positive staining and (F) the histopathology sum scores. Results combining 2–3 independent experiments are shown. Individual mouse data are reported according to sexes with bars/errors indicating either geometric mean ± geometric SD (viral titers) or mean ± SD (pathology scores and NP staining). Dotted line represents RT‐qPCR detection limit. Significance between female and male mice infected with the same virus at the same time point was determined by Mann‐Whitney test. Viral titers were log transformed before the statistical analysis. *p < 0.05.

No significant sex differences were observed in nasal or lung hACE2 expression level despite female mice tended to show higher expression than male mice (Supporting Information Figure 1). We then conducted sex‐specific analysis to reveal the pathology patterns of nasal cavity and lung induced by individual SARS‐CoV‐2 variants. While no significant sex‐biased differences were noted for viral RNA level on either 3‐ or 5‐ dpi (Figure 2A,B), male mice generally tended to have more NP positive foci in nasal cavity and lung than female mice across all variants (Figure 2C,D). 614 G infection was the exception, in which female mice had more NP‐stained viral foci in nasal cavity on 5‐dpi (Figure 2C) and more severe nasal histopathology on 3‐dpi than male mice (Figure 2E). When comparing lung histopathology across all variants, Delta‐infected males generally exhibited more type II pneumocyte hyperplasia and more frequent and severe interstitial/perivascular edema than Delta‐infected female mice and other variants‐infected groups (Supporting Information Table 2).

**Figure 3:**
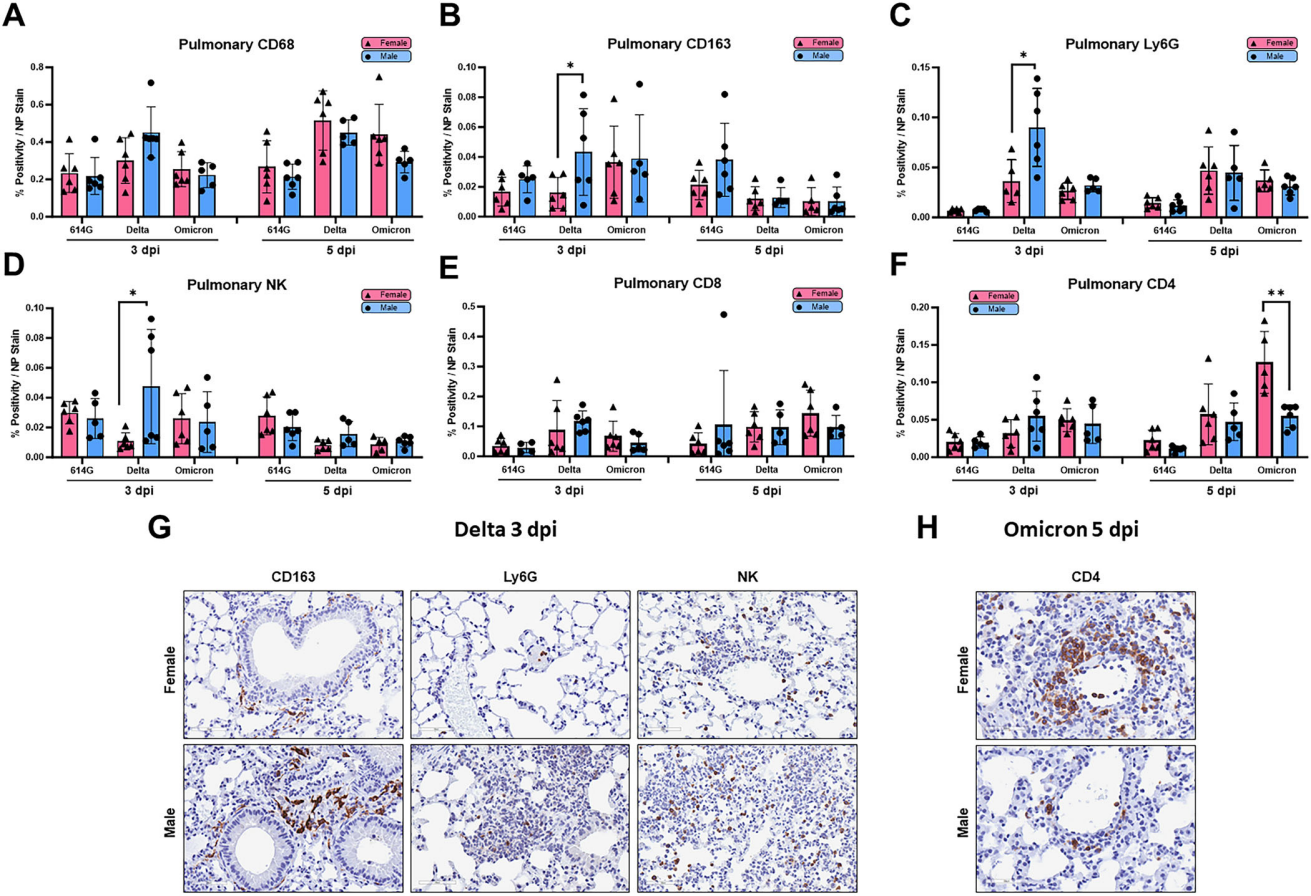
Sex‐specific immune cell recruitment in infected mouse lungs across SARS‐CoV‐2 variants. Lung histologic sections from 614 G, Delta or Omicron‐infected mice on 3‐ and 5‐days post infection (dpi) were immunostained. (A‐F) The percent positive immune cells normalized to percent viral nucleocapsid staining within three defined regions of interest per sample. Representative images of immunostained pulmonary tissue: (G) Delta‐infected females and males on 3‐dpi; (H) Omicron‐infected females and males on 5‐dpi. Results combining two independent experiments are shown. Individual mouse data are plotted according to sexes with bars/errors indicating mean ± SD. Significance between female and male mice infected with the same virus at the same time point were determined by Mann‐Whitney test. *p < 0.05, **p < 0.01.

Lung IHC staining revealed that Delta infection resulted in significantly greater pulmonary recruitment of CD68+ macrophages as well as Ly6G+ neutrophils than the other two variants at both timepoints (Supporting Information Figures 2A,C). When sex‐specific analysis was performed, Delta‐infected males showed significantly increased recruitment of CD163+ “M2” macrophages, Ly6G+ neutrophils and NKR‐P1C + NK cells than Delta‐infected females on 3‐dpi (Figure 3B‐D,G), indicating a prominent innate immune response in males on early onset of infection. On the other hand, pulmonary infiltrated CD8+ and CD4 + T lymphocytes were the highest in Omicron‐infected mice on 5‐dpi, followed by Delta, and was lowest in 614G‐infected animals (Supporting Information Figure 2E,F). Of note, female mice had significantly increased lung CD4 + T cell infiltration than males on 5‐dpi after Omicron infection (Figure 3F,H), suggesting a more pronounced but delayed humoral immune response in females. These results indicate that sex‐oriented infection patterns in nasal cavity and lung are specific for individual SARS‐CoV‐2 variants.

### 3.3 Sex‐Specific Analysis of Pulmonary Inflammation

Consistent with viral load and tissue pathology, lung inflammation as measured by pulmonary cytokines varied significantly across different SARS‐CoV‐2 variants (Supporting Information Figure 3). Overall, Delta‐infected animals had greater lung secretion of IL‐10, IL‐6, MCP‐1 and MIP‐1α than 614G‐ and Omicron‐infected animals (Supporting Information Figure 3A,C,F,G). However, Omicron‐infected animals had significantly elevated IP‐10 secretion than Delta and 614G‐infected mice especially on 5‐dpi (Supporting Information Figure 3E).

**Figure 4:**
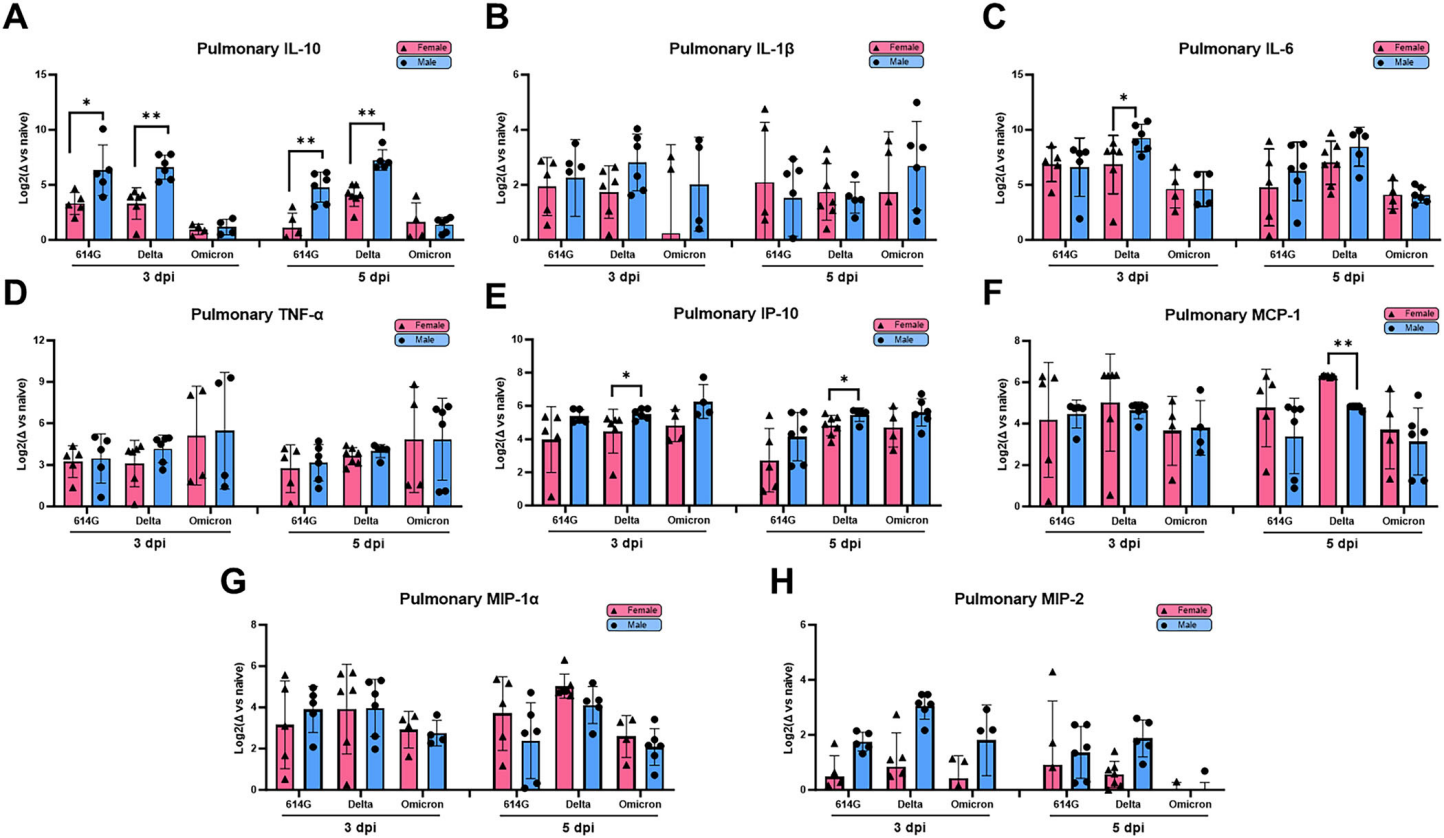
Sex‐specific pulmonary cytokine measurements across SARS‐CoV‐2 variants. (A‐H) Pulmonary cytokines on 3‐ or 5‐days post infection (dpi) normalized to naïve animals as Log2 fold changes. Results combining three independent experiments are shown. Individual mouse data are plotted according to sexes with bars/errors indicating mean ± SD. Significance between female and male mice infected with the same virus at the same time point were determined by Mann‐Whitney test. *p < 0.05, **p < 0.01.

When analyzing cytokine levels by sex, males infected with 614 G, or Delta consistently showed significantly elevated IL‐10 levels than females on both 3‐ and 5‐dpi (Figure 4A). Additionally, Delta‐infected males had greater pulmonary secretion of IL‐6 on 3‐dpi and IP‐10 on 3‐ and 5‐dpi than Delta‐infected females (Figure 4C,E). In contrast, Delta‐infected females had significantly higher MCP‐1 secretion in lung than Delta‐infected males on 5‐dpi (Figure 4F). No significant sex differences were observed for IL‐1β, TNF‐*α*, MIP‐1*α*, or MIP‐2 across all SARS‐CoV‐2 variants, however these inflammatory cytokines were trending higher in males (Figure 4B,D,G,H).

The pulmonary cytokine profiles confirm the histopathology observations that male and female mice mount distinct immune responses to infection with SARS‐CoV‐2 variant.

### 3.4 Sex‐Specific Spatial Transcriptomic Analysis of Delta‐Infected Lungs

**Figure 5:**
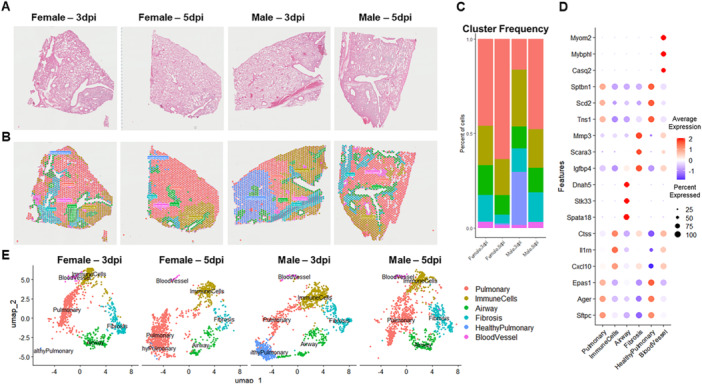
Sex‐specific spatial transcriptomics analysis in Delta‐infected mouse lungs. Lungs from Delta‐infected female and male mice on 3‐ and 5‐days post infection (dpi) were processed for spatial transcriptomics. (A). H&E staining. (B) Spatial plots with dimensional reduction clustering overlay with individual clusters coded by color and each dot representing a capture spot. (C) Relative expression frequency of gene clusters across each sample in a bar plot. (D) Conserved gene markers in identified clusters shown in a dot plot with average gene expression denoted by a color (red = upregulated, blue = downregulated) and percent expression denoted by the size of a dot. (E) UMAP plots for individual samples with 6 identified major clusters. Each dot in the UMAP plots represents one of 1,317 ± 136 capture spots per sample shown in B and color denotes corresponding gene expression cluster.

Because Delta‐infected lungs exhibited more pronounced sex‐specific immune response, we performed spatial transcriptomics on H&E‐stained lung sections to further elucidate sex differences after Delta infection (Figure 5A). Sequenced libraries from each tissue section were aligned to a reference genome and mapped back to the tissue section to create a spatially resolved gene expression map (Figure 5B). Six spatial clusters were identified, including Pulmonary, ImmuneCell, Airway, Fibrosis, HealthyPulmonary, and BloodVessel. Regardless of sex, the most abundant clusters were associated with pulmonary genes and immune response (Figure 5C). Clusters were annotated by identifying highly upregulated, conserved genes in each cluster (Figure 5D), as well as correlation to tissue morphology. Visual examination of UMAP plots reveals distinct patterns across each Delta‐infected lung with an apparent shift in pulmonary‐ and immune cell‐associated tissue spots between males and females (Figure 5E).

**Figure 6:**
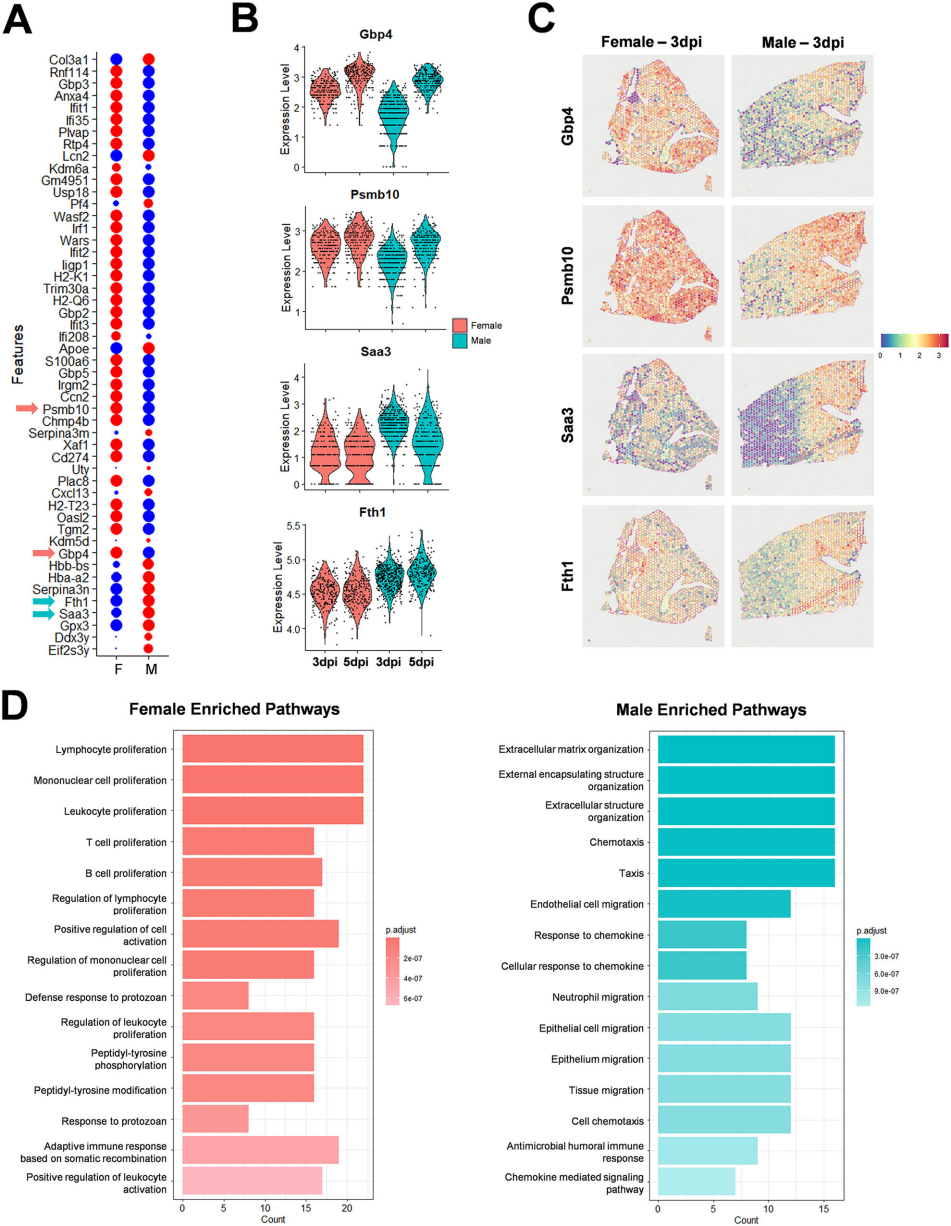
Differential analysis of spatial transcriptomics identified gene markers within the lung immune cell clusters. Lungs from Delta‐infected female and male mice on 3‐ and 5‐days post infection (dpi) were analyzed by spatial transcriptomics. (A) Top 50 genes that are significantly associated with female (left) or male (right) ‘ImmuneCell’ cluster (presented in Figure 5) in a dot plot with average gene expression denoted by a color (red = upregulated, blue = downregulated) and percent expression denoted by the size of a dot. (B) Gene expression of Gbp4, Psmb10, Saa3, or Fth1 in violin plot. (C) Spatially mapped gene expression of Gbp4, Psmb10, Saa3, or Fth1 on 3‐dpi. (D) Top 15 enriched pathways for female (left) and male (right) samples identified by gene ontology analysis.

To further elucidate sex‐specific differences within the ImmuneCell population in Delta‐infected lung tissues, the top 50 differentially expressed genes were identified (Figure 6A). Among these genes, the top 10 genes that were differentially enriched in Delta‐infected males or females were revealed (Figure 6B and Supporting Information Figure 4). Gbp4 (guanylate binding protein 4) and Psmb10 (proteasome subunit beta type‐10) were two representative genes that were preferentially upregulated in the lungs of Delta‐infected females as early as 3 dpi (Figure 6B,C). In contrast, Delta‐infected males had Saa3 (serum amyloid A3) and Fth1 (ferritin heavy chain) elevated early in lung than females (Figure 6B,C). The biological pathways associated with the top 10 sex‐biased genes were identified by gene ontology, which revealed that immune cell proliferation and activation were the highest enriched pathways in Delta‐infected females, whereas extracellular matrix production, chemokine signaling, and cell chemotaxis were highly associated with Delta‐infected males (Figure 6D).

The results of the transcriptomic analysis of Delta‐infected lung sections indicate that male and female mice had different immune pathways activated in response to SARS‐CoV‐2 infection.

## 4 Discussion

Male and female often respond differently toward infectious and noninfectious inflammatory diseases [29]. For instance, females have the higher prevalence of autoimmune disease but often mount stronger adaptive immune responses and have greater vaccine efficacy toward infections than males [29, 30]. In contrast, males are often reported to experience higher susceptibility to respiratory diseases with increased inflammation and pathological findings compared to females [31]. Similar observations were reported for COVID‐19, wherein men reportedly have experienced greater disease severity than women including higher infection rate, imbalanced immune response, more intensive care unit admission and higher fatality, especially during the early pandemic [8, 10, 13, 14, 15]. However, different SARS‐CoV‐2 variants with distinct antigenic and pathogenic properties may also alter the sex‐bias in the disease outcomes. For example, during the Alpha wave, a UK study reported that women had an increased risk of hospital admission and a modest increased risk of mortality [32]. Conversely, other studies reported higher infection rates in men compared to women during the first five waves of the pandemic, with especially more men contracting the Delta variant and exhibiting increased mortality than women [33, 34, 35, 36]. During the Omicron‐prevalent sixth wave, however, women were reportedly at higher risks of infection and were slower to recover than men [36, 37, 38]. These epidemiology studies were limited to specific population(s) studied and were confounded by race, ethnicity, age, sex, comorbidity, etc. Although the epidemiological data agree on that sex is a risk factor for COVID‐19, they cannot pinpoint which sex is more susceptible to SARS‐CoV‐2 infection and if sex‐biased differences are specific for individual variants.

To address these questions, we conducted the current study by infecting age‐matched female and male K18‐hACE2 transgenic mice with representative SARS‐CoV‐2 variants – 614 G, Delta and Omicron BA.1. Our study shows Delta causes most severe disease in the nasal cavity and lung, followed by 614 G, then Omicron BA.1 which results in the least damage in the respiratory tract of K18‐hACE2 mice. These observations are consistent with the epidemiological reports that the Delta predominant wave had the overall greatest disease severity in terms of clinical manifestations, hospitalization rates, ICU admissions and in‐hospital mortality; while patients infected with Omicron variant had drastically reduced disease severity and significantly lower risk of hospitalization than the cases in the prior waves [32, 33, 35, 37, 38, 39, 40, 41]. Furthermore, Delta variant elicited the most sex‐biased outcomes in K18‐hACE2 mice, which infected males showed increased respiratory recruitment of neutrophils, NK cells, and CD163+ macrophages along with sustained pulmonary inflammatory cytokines such as IL‐10, IP‐10, and IL‐6, whereas infected females had significantly elevated pulmonary MCP‐1. Cytokine release syndrome (or cytokine storm) is a severe complication of COVID‐19 and is associated with acute respiratory distress syndrome and multiorgan dysfunction [42, 43, 44]. Increased COVID‐19 disease severity is reportedly associated with progressively elevated of inflammatory cytokines such as IL‐10, IP‐10, IL‐6, or MCP‐1 [43, 44]. Additionally, MCP‐1 level in critically ill COVID‐19 patients has been found predictive for the duration of mechanical ventilation usage and ICU stay [43]. Consistent with human clinical studies, we demonstrated that elevated pulmonary inflammation in combination with sustained virus presence resulted in more severe pathological changes and damage in the lungs of Delta infected animals than those infected with Omicron variant.

Using spatial transcriptomics, we identified that in the lungs of Delta‐infected male mice genes associated with extracellular matrix production, chemokine signaling, and cell chemotaxis were preferentially upregulated, for example, Saa3 and Fth1. Saa3 is known to modulate macrophage and neutrophil function during acute‐phase inflammation [45], and Fth1 is reportedly a marker of severe pulmonary inflammation and cellular damage and can promote a fibrotic response [46, 47]. Increased expression of Saa3 and Fth1 in the lungs of Delta‐infected male mice indicates more pronounced inflammation and severe tissue damage. In contrast, Delta‐infected female mice had upregulated expression of Psmb10 (MHC‐Class I dependent antigen presentation) [48] and Gbp4 (inflammasome dependent pathogen clearance) than infected males [49]. These genes are related to humoral immune response and antiviral interferon signaling, suggesting Delta infected males may have a reduced capability for antigen presentation and virus clearance than female mice. A recent study by Rizvi et al. has also reported that Delta‐infected male mice had increased thymic atrophy with impaired T cell maturation than infected females [50]. Clinical studies have also described that decreased CD4 + T cells or poor CD8 + T cell activation is associated with male patients with more severe COVID‐19 [10, 12].

In summary, this study demonstrates the utility of the K18‐hACE mouse model for studying sex differences in SARS‐CoV‐2 virulence and host immune response. The results obtained show that male and female mice elicit variable responses with respect to specific variants encountered, similar to humans. The differential gene expression patterns observed in our study serve as a starting point for elucidating differences in male and female immune responses to SARS‐CoV‐2. Understanding these differences is important for developing sex‐specific therapeutic approaches and vaccines.

## Author Contributions

Hang Xie conceived the study, acquired the funding and designed the mouse experiments. Hang Xie and Kelly E. Mercer supervised the investigations. Hang Xie, Weichun Tang, Insung Kang, Martina Kosikova, Hyung‐Joon Kwon and Uriel Ortega‐Rodriguez performed mouse experiments and collected tissues. Weichun Tang, Insung Kang, Martina Kosikova and Hyung‐Joon Kwon processed tissues and performed viral RNA extraction. Weichun Tang conducted RT‐qPCR and data analysis. Uriel Ortega‐Rodriguez, Insung Kang, Martina Kosikova, Weichun Tang and Hyung‐Joon Kwon conducted tissue cytokines and data analysis. Jennifer H. Hanks and Lana Elkins conducted tissue pathology and IHC. Jennifer H. Hanks conducted histopathology evaluation. Elysia A. Masters and Kelly E. Mercer conducted IHC quantification and analysis. Elysia A. Masters conducted spatial transcriptomics. Elysia A. Masters and Binsheng Gong conducted spatial transcriptomic analysis. Elysia A. Masters and Hang Xie drafted the manuscript. Hang Xie, Elysia A. Masters and Kelly E. Mercer revised the manuscript. All authors reviewed the manuscript and agreed on the submission.

## Ethics Statement

The conduction of mouse experiments and collection of mouse tissues were performed according to the protocols approved by FDA White Oak Vivarium IACUC.

## Conflicts of Interest

The authors declare no conflicts of interest.

## Supporting information

JMV‐25‐24108 Suppl Inform.

## Data Availability

The data that support the findings of this study are available from the corresponding authors on reasonable request.
