# Supplementary material for: Dissecting Sex‐Specific Pathology in K18‐hACE2 Transgenic Mice Infected With Different SARS‐CoV‐2 Variants
Source: J Med Virol. 2025 Jul 21;97(7):e70506. doi: 10.1002/jmv.70506 (PMC12277941; doi:10.1002/jmv.70506)
Supplement: Supplementary file 1 — JMV‐25‐24108 Suppl Inform. [file JMV-97-e70506-s001.zip › JMV-25-24108_Suppl_Inform.docx]

Supplemental Information

Dissecting Sex-Specific Pathology in K18-hACE2 Transgenic Mice Infected with Different SARS-CoV-2 Variants

Elysia A. Masters^1†^, Weichun Tang^2†^, Insung Kang^2^, Martina Kosikova^2^, Jennifer H. Hanks^3,4^, Lana Elkins^3^, Hyung-Joon Kwon^2^, Uriel Ortega-Rodriguez^2^, Binsheng Gong^5^, Kelly E. Mercer^1^*, Hang Xie^2^*.

^1^Division of Systems Biology, National Center for Toxicological Research, United States Food and Drug Administration, Jefferson, AR 72079 USA;

^2^Division of Viral Products, Office of Vaccines Research and Review, Center for Biologics Evaluation and Research, United States Food and Drug Administration, Silver Spring, MD 20993 USA;

^3^Toxicologic Pathology Associates, Jefferson, AR, 72079 USA;

^4^Division of Neurotoxicology, National Center for Toxicological Research, United States Food and Drug Administration, Jefferson, AR 72079 USA;

^5^Division of Bioinformatics and Biostatistics, National Center for Toxicological Research, United States Food and Drug Administration, Jefferson, AR 72079 USA

^†^These authors have contributed equally to this work and share first authorship.

*Correspondence to: Hang Xie (current email: Hang.Xie@cdc.hhs.gov); Kelly E. Mercer (Kelly.Mercer@fda.hhs.gov).

## Supplemental Table 1. Nasal cavity histopathology evaluation^*^.

| **Variants** | **N** | **dpi^#^** | **Inflammatory Infiltrates** | **Intranasal Exudate** | **Mucosal Degeneration** |
| --- | --- | --- | --- | --- | --- |
| Naïve | 8 | 0 | 0.00 ± 0.00 | 0 .00 ± 0.00 | 0 .00 ± 0.00 |
| 614G | 12 | 3 | 1.91 ± 0.19^b^ | 0.16 ± 0.57^a^ | 1.00 ± 0.85^b^ |
|  | 12 | 5 | 1.16 ± 0.38 | 0.33 ± 0.65 | 0.41 ± 0.51^e^ |
| Delta | 20 | 3 | 1.82 ± 0.59^b^ | 1.00 ± 0.64^b^ | 1.82 ± 1.11^c^ |
|  | 17 | 5 | 1.23 ± 0.50 | 0.44 ± 0.71 | 1.29 ± 1.45^f^ |
| Omicron | 17 | 3 | 1.18 ± 0.67^a^ | 0.06 ± 0.24^a^ | 0.11 ± 0.33^a^ |
|  | 18 | 5 | 0.97 ± 0.67 | 0.00 ± 0.00 | 0.00 ± 0.00^e^ |

^*^Age-matched female and male K18-hACE2 mice were infected with SARS-CoV-2 614G, Delta or Omicron variant. Nasal cavity was collected for histopathology. Lesions were evaluated in a blind fashion by a board-certified veterinary pathologist and graded for severity according to the following scale: 1 (minimal), 2 (mild), 3 (moderate) or 4 (marked). Group scores are presented in means ± SD. Significance was determined by 2-way ANOVA with Holm-Sidak post hoc analysis. ^#^dpi: days post infection. For strain differences at 3 dpi, P < 0.05, a<b<c; for strain differences at 5 dpi, P < 0.05, e<f.

## Supplemental Table 2. Lung histopathology evaluation^*^.

| Category Score# | Uninfected naive  (n=12) | 614G | | Delta | | Omicron | |
| --- | --- | --- | --- | --- | --- | --- | --- |
|  |  | 3-dpi^#^  (n=18) | 5-dpi  (n=18) | 3-dpi  (n=24) | 5-dpi  (n=27) | 3-dpi  (n=24) | 5-dpi  (n=24) |
| Interstitial Infiltrates | 0.17  (0.41) | 1.36 (0.58)^a^ | 1.08 (0.42)^e^ | 1.81 (0.62)^b^ | 2.14 (0.38)^g,h^ | 1.31 (0.58)^a^ | 1.50 (0.70)^f^ |
| Alveolar Infiltrates | 0.00  (0.00) | 0.66 (0.48)^a^ | 0.61 (0.67)^e^ | 1.56 (0.79)^b^ | 1.88 (0.64)^f^ | 0.64 (0.92)^a^ | 0.81 (0.67)^e^ |
| Hyperplasia, type II pneumocyte | 0.00  (0.00) | 0.00 (0.00) | 0.00 (0.00) | 0.00 (0.00) | 0.14 (0.36) | 0.00  (0.00) | 0.00 (0.00) |
| Alveolar Macrophages | 0.17  (0.41) | 0.11 (0.32) | 0.11 (0.32)^e^ | 0.29 (0.55) | 1.33 (0.87)^g,h^ | 0.68  (0.72) | 0.81 (0.84)^f^ |
| Vasculitis, perivasculitis | 0.00  (0.00) | 1.22 (0.55)^a^ | 1.27 (0.57)^e^ | 2.52 (0.54)^c^ | 2.75 (0.40)^g^ | 1.85 (0.54)^b^ | 1.97 (0.74)^f^ |
| Edema, Alveolar | 0.00  (0.00) | 0.00 (0.00) | 0.00 (0.00) | 0 .08 (0.28) | 0.26 (0.53) | 0.08  (0.28) | 0.04  (0.20) |
| Erythrocytes, intraalveolar | 0.83  (1.33) | 0.11 (0.47) | 0.44 (0.70) | 0.33 (0.56) | 0.37 (0.88) | 0.37  (0.77) | 0.25  (0.44) |
| Congestion | 0.50  (0.84) | 0.44 (0.85) | 0.27 (0.66) | 0.25 (0.61) | 0 .22 (0.64) | 0.21  (0.58) | 0.25  (0.61) |
| Edema, interstitial/ perivascular | 0.00  (0.00) | 0.28 (0.66) | 0.11 (0.47)^e^ | 0.58 (0.83) | 1.00 (0.64)^f,h^ | 0.25  (0.44) | 0.08 (0.28)^e^ |
| SARS-CoV-2, IHC | 0.00  (0.00) | 1.55 (0.71)^a^ | 1.61 (1.33)^e^ | 3.21 (1.02)^b^ | 2.63 (1.27)^f^ | 2.16 (1.04)^a^ | 1.87 (1.29)^e^ |

^*^Age-matched female and male K18-hACE2 mice were infected with SARS-CoV-2 614G, Delta or Omicron variant. Lungs was collected for histopathology. Lesions were evaluated in a blind fashion by a board-certified veterinary pathologist and graded for severity according to the following scale: 1 (minimal), 2 (mild), 3 (moderate) or 4 (marked). Group scores are presented as means (SD). Significance was determined by 2-way ANOVA with Holm-Sidak post hoc analysis. ^#^dpi: days post infection. For strain differences on 3-dpi, P < 0.05 a<b<c; for strain differences on 5-dpi, P < 0.05 e<f<g. h indicates P <0.01 for differences between 3- and 5-dpi within strains.

Supplemental Figure 1. Comparison of tissue-specific hACE2 expression between male and female mice. The hACE2 copy numbers in nasal turbinate and lung tissues of age-matched male and female K18-hACE2 mice were quantitated by real-time PCR. Individual mouse data are plotted with bars/errors indicating mean ± SD. No significant difference was observed between male vs female mice by one-way nonparametric ANOVA.


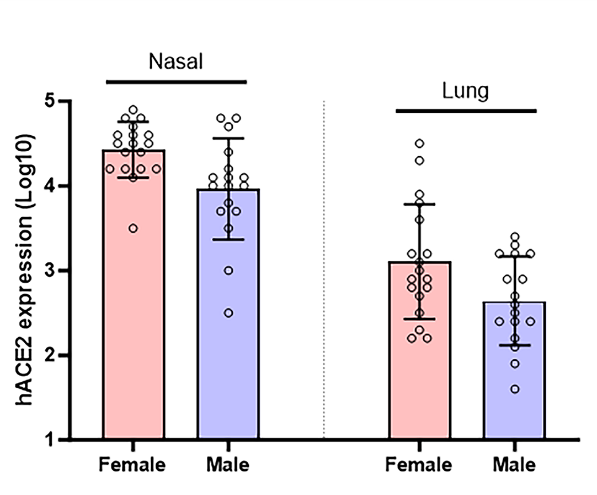


Supplemental Figure 2. Total quantification of pulmonary immune cell recruitment across SARS-CoV-2 variants. (A-F) Lung histologic sections from 614G, Delta or Omicron-infected mice on 3- or 5-days post infection (dpi) were immunostained for viral nucleocapsid (NP), CD68, CD163, Ly6G, NKR-P1C, CD8 or CD4, respectively. The percent positive immune cells were quantified using Aperio ImageScope software and normalized to percent NP staining within 3 defined regions of interest per sample. Individual mouse data are plotted with bars/errors indicating mean ± SD. Significance was evaluated by two-way ANOVA with Tukey’s post-hoc test for multiple comparisons. *p < 0.05, **p < 0.01, ***p < 0.001, ****p <0.0001.


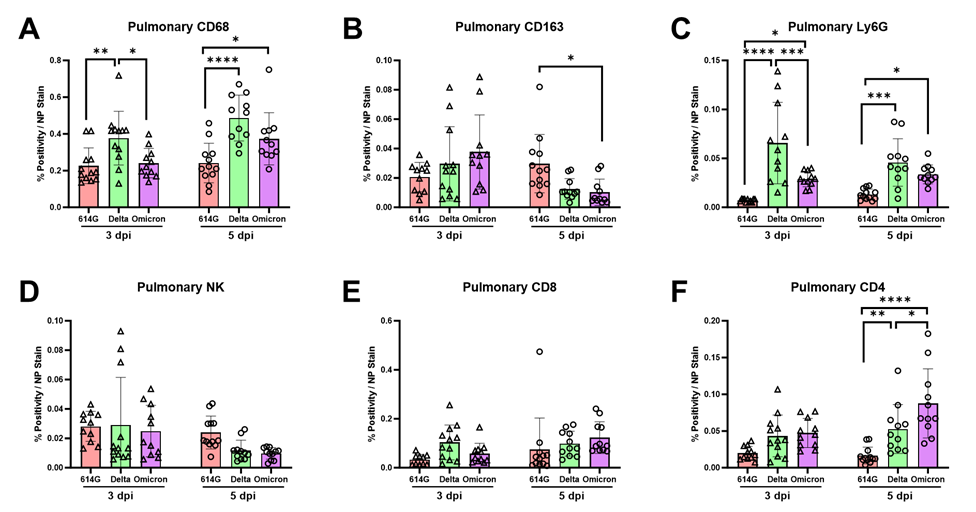


**Supplemental Figure 3. Total pulmonary cytokine measurements across SARS-CoV-2 variants.** (A-H) Pulmonary cytokines in 614G, Delta or Omicron-infected mice on 3-or 5-days post infection (dpi) were measured by multiplexed antibody detection and were normalized to naïve animals as log2 fold changes. Individual mouse data are plotted with bars/errors indicating mean ± SD. Significance was evaluated by two-way ANOVA with Tukey’s post-hoc test for multiple comparisons. *p < 0.05, **p < 0.01, ***p < 0.001, ****p <0.0001.

**
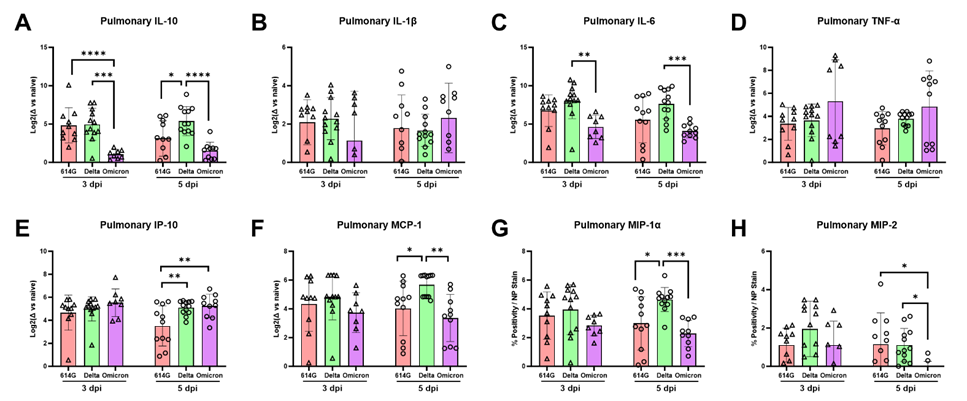
**

**
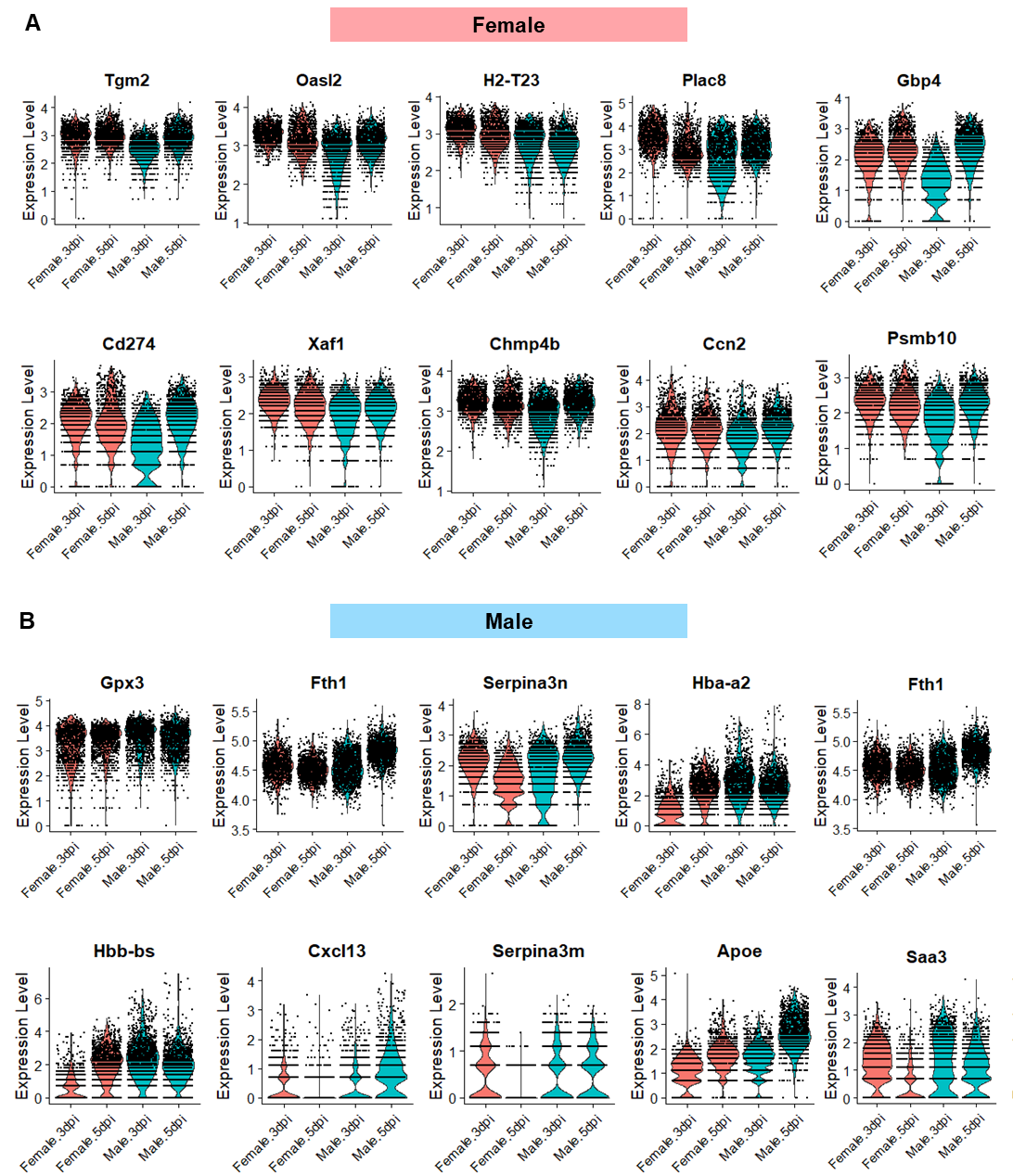
Supplemental Figure 4. Top 10 differentially expressed genes in male and female lungs infected with Delta SARS-CoV-2.** Differential analysis of spatial transcriptomics data identified gene markers within the immune cell cluster that are significantly associated with female vs male samples. The top 10 differentially expressed genes between female (A, top) and male (B, bottom) ‘ImmuneCell’ cluster were displayed by Violin plot. Data represents expression level across all clusters in each sample.
