# Supplementary figures and images for: Dissecting Sex‐Specific Pathology in K18‐hACE2 Transgenic Mice Infected With Different SARS‐CoV‐2 Variants
Source: J Med Virol. 2025 Jul 21;97(7):e70506. doi: 10.1002/jmv.70506 (PMC12277941; doi:10.1002/jmv.70506)

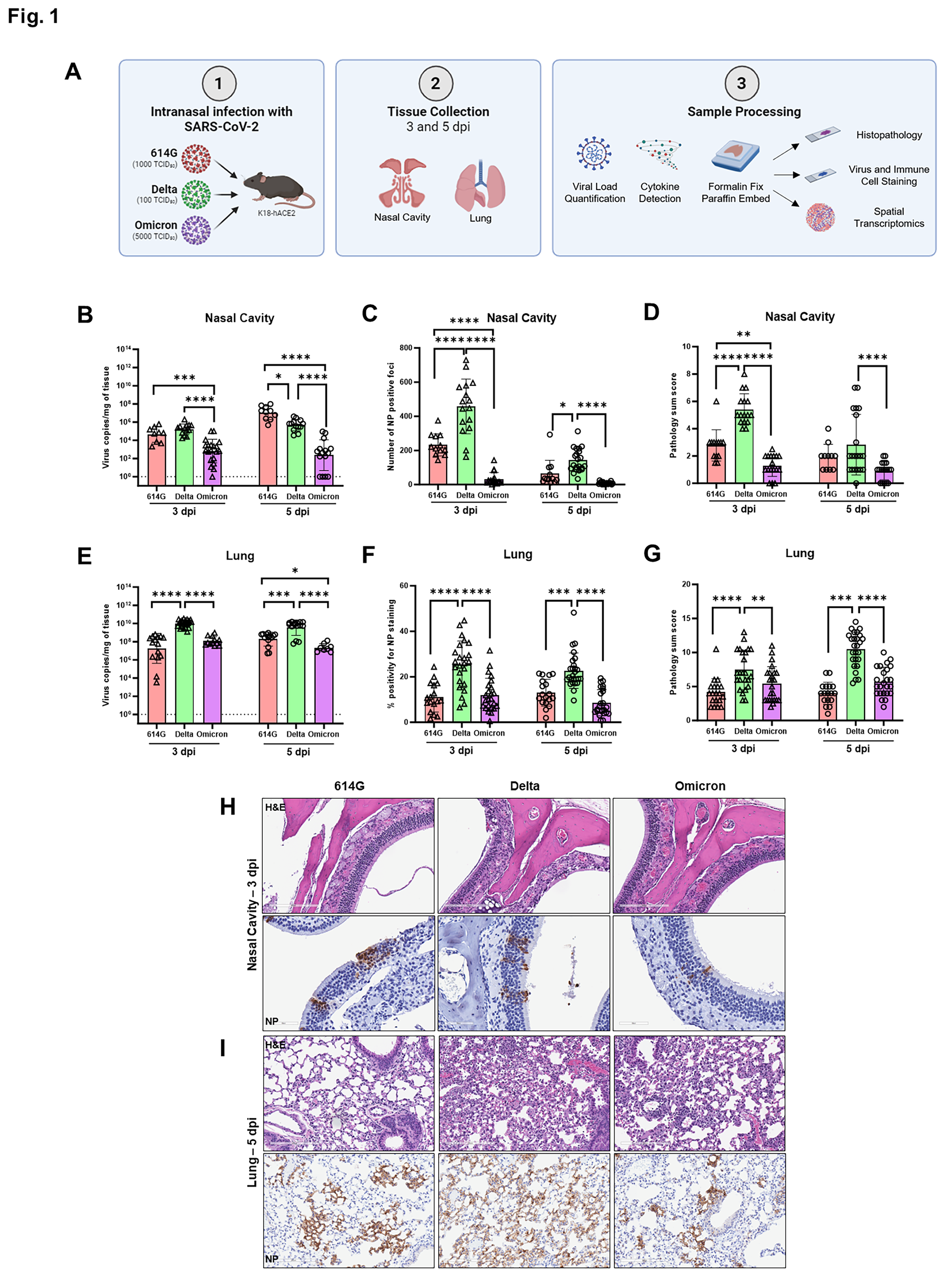

Supplement: Supplementary file 1 — JMV‐25‐24108 Suppl Inform. [file JMV-97-e70506-s001.zip › Figure_1.tif]

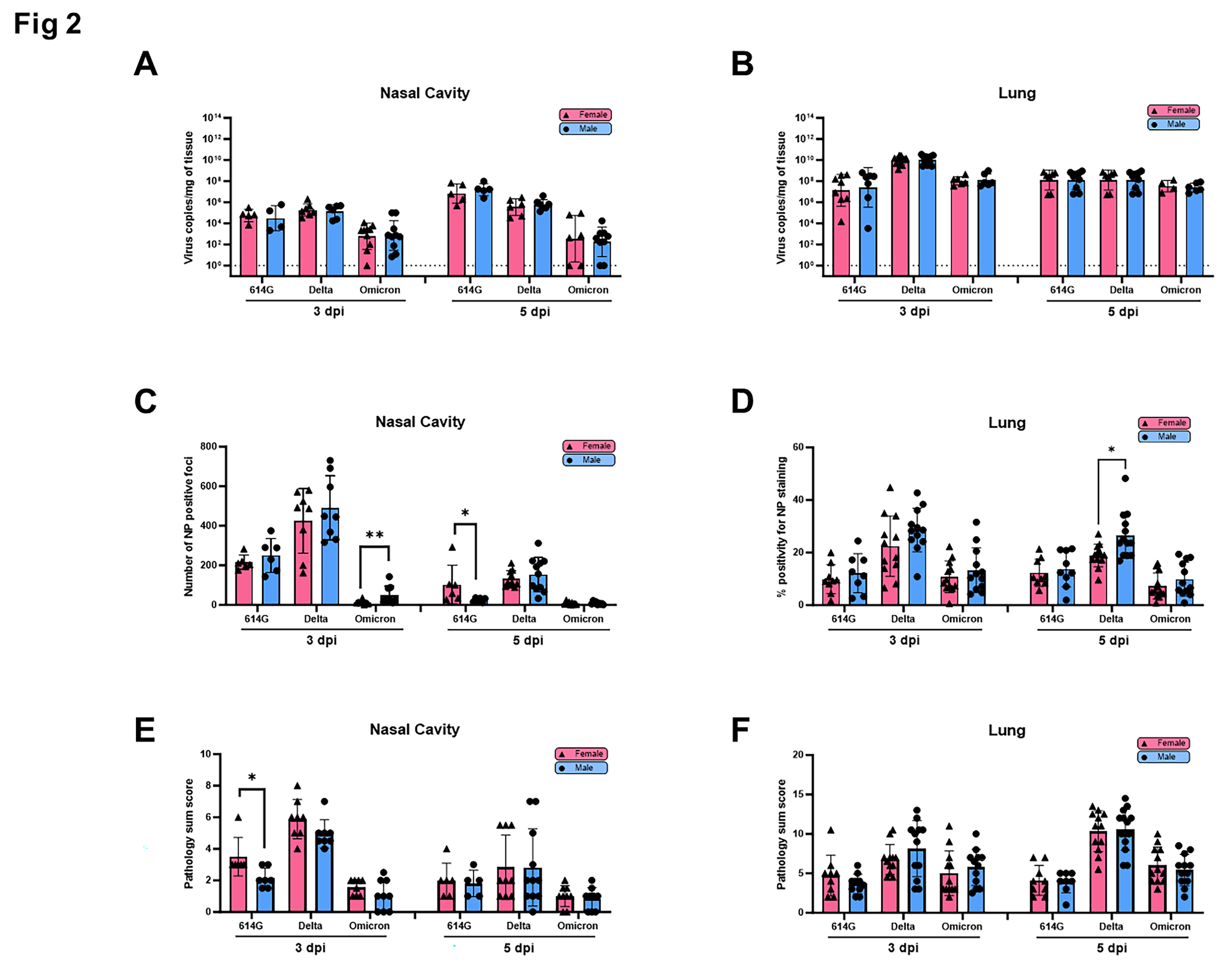

Supplement: Supplementary file 1 — JMV‐25‐24108 Suppl Inform. [file JMV-97-e70506-s001.zip › Figure_2.tif]

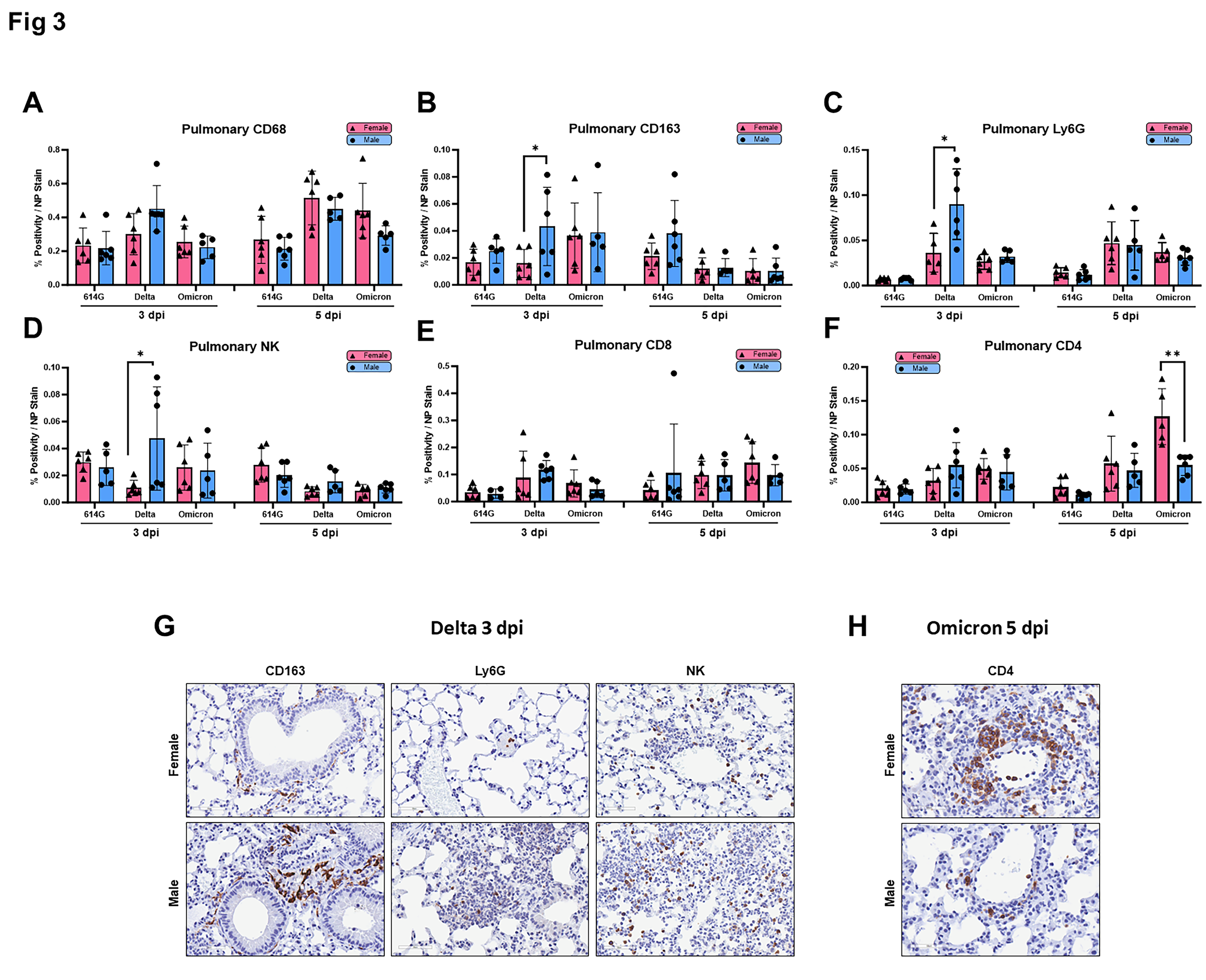

Supplement: Supplementary file 1 — JMV‐25‐24108 Suppl Inform. [file JMV-97-e70506-s001.zip › Figure_3.tif]

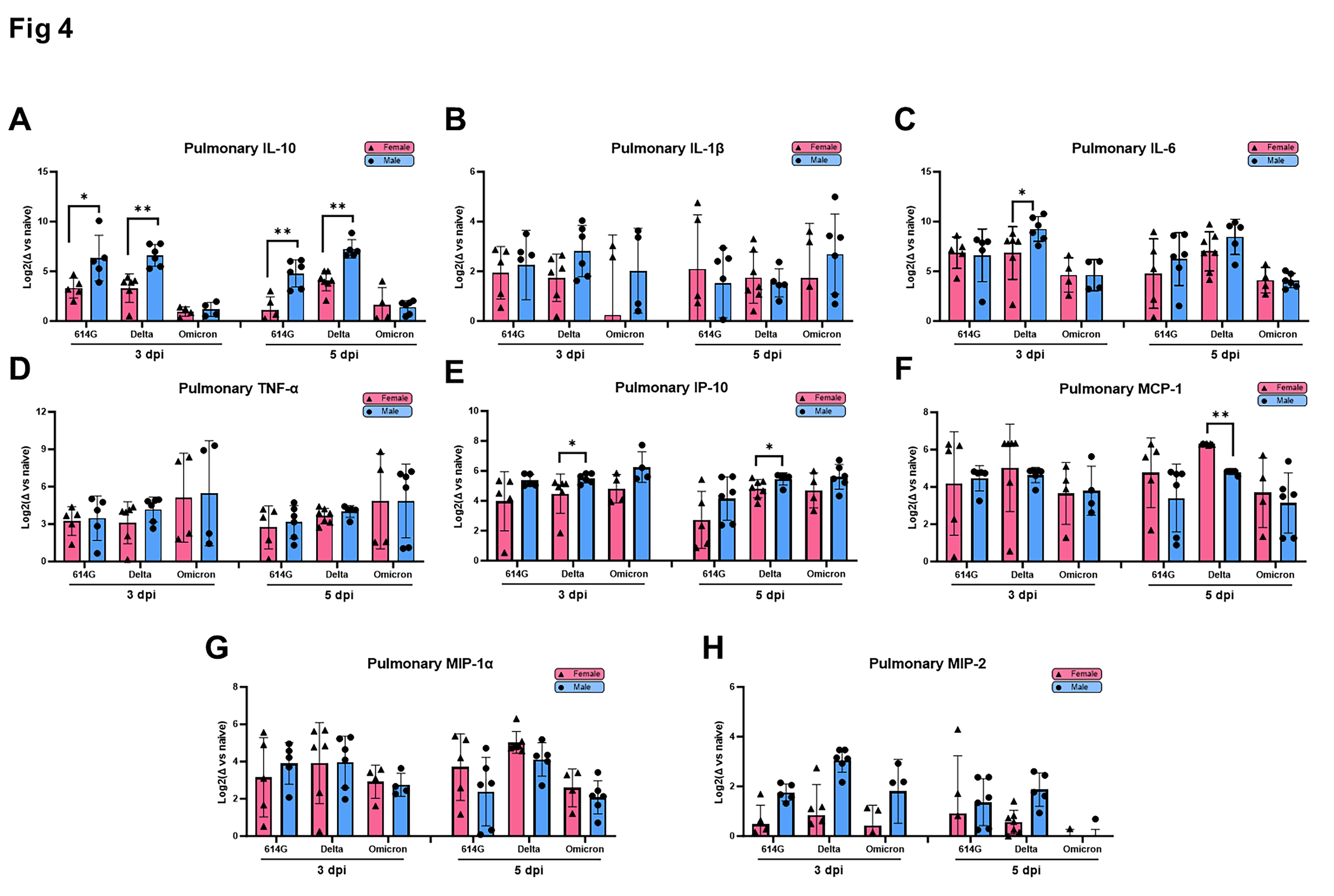

Supplement: Supplementary file 1 — JMV‐25‐24108 Suppl Inform. [file JMV-97-e70506-s001.zip › Figure_4.tif]

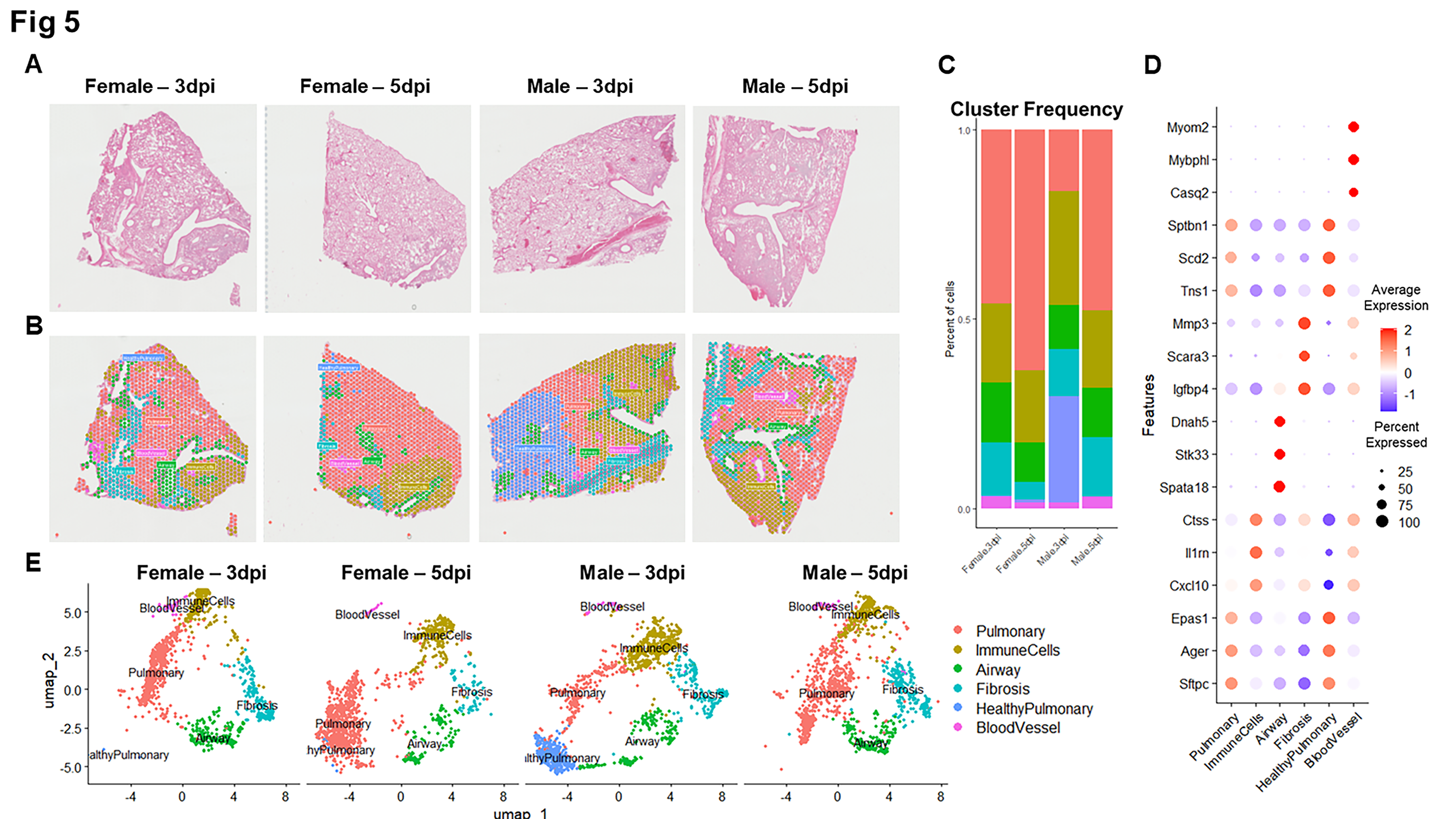

Supplement: Supplementary file 1 — JMV‐25‐24108 Suppl Inform. [file JMV-97-e70506-s001.zip › Figure_5.tif]

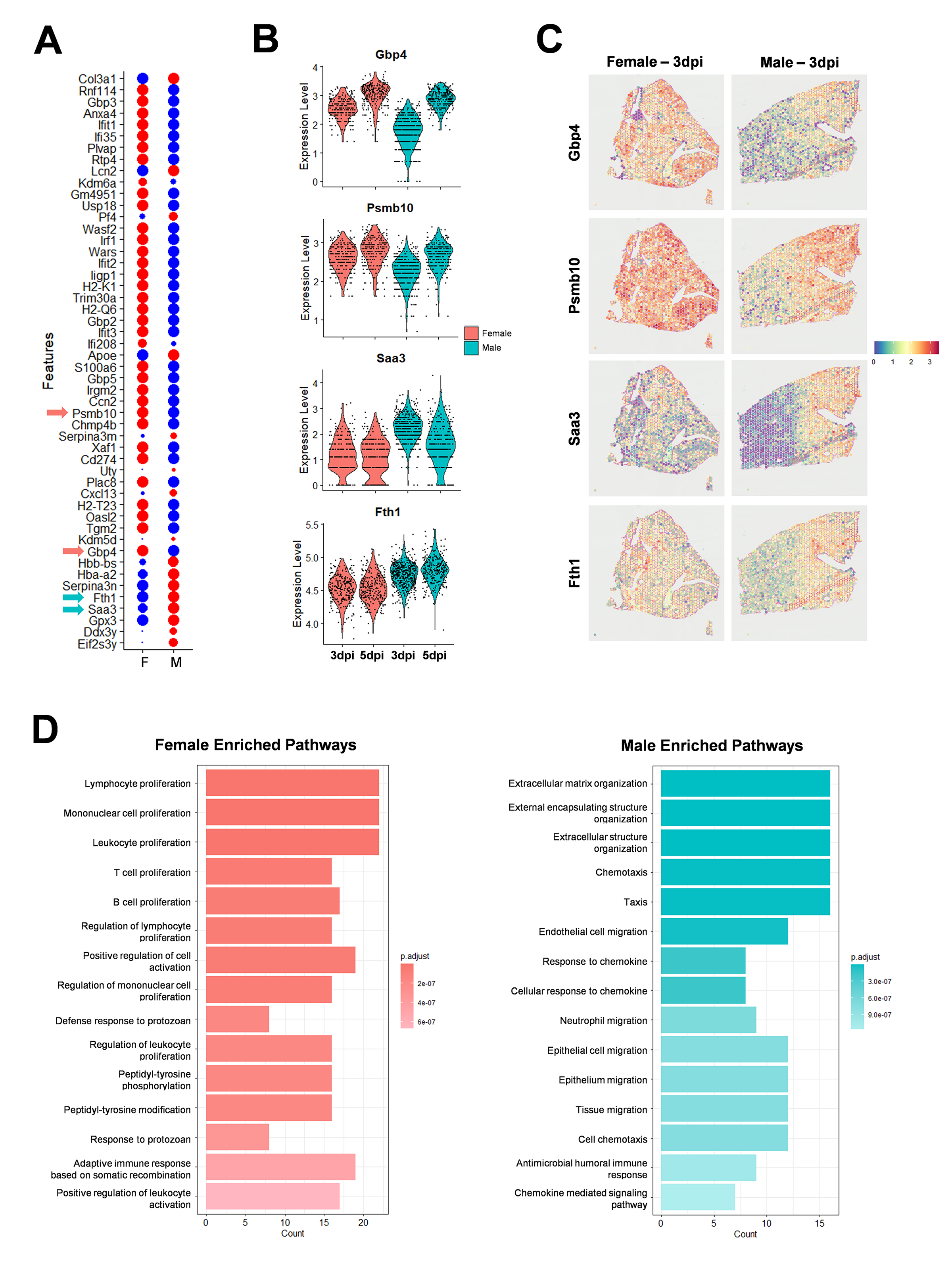

Supplement: Supplementary file 1 — JMV‐25‐24108 Suppl Inform. [file JMV-97-e70506-s001.zip › Figure_6.tif]
